# Development and Validation of a Complexometric and Potentiometric Titration Method for the Quantitative Determination of Zinc Pyrithione in Shampoo

**DOI:** 10.1155/2021/6661744

**Published:** 2021-01-06

**Authors:** Greciel E. Egurrola, Angela P. Mazabel, Johnbrynner García

**Affiliations:** Belcorp Research and Development, Tocancipá 251017, Colombia

## Abstract

Most of the pharmaceutical and cosmetic products used for the treatment of dandruff have zinc pyrithione as an active ingredient; therefore; quantifying this component becomes necessary. The purpose of this study was the validation of two simple and fast methodologies in the quantification of zinc pyrithione for shampoo quality control to guarantee consumer safety. The first method comprised a manual complexometric titration, and the second comprised a potentiometric titration performed with an automatic titrator, obtaining sensitivity values of 0.0534% and 0.0038%, respectively, precision expressed in RSD% values below than 1%, and accuracy in recovery percentage greater than 99%. Additionally, both methods were robust when subjected to significant changes in working conditions (temperature and pH) and were selective even in the presence of interferences and degradation products. Finally, the methodologies were adequate to ensure the quality of shampoo to ensure the safety of consumers.

## 1. Introduction

Zinc pyrithione (ZnPT) is an organometallic compound with chemical formula C_10_H_8_N_2_O_2_S_2_Zn that has bactericidal and fungicidal activity. It is the antifungal agent most used in shampoos for control of dandruff due to its versatility. ZnPT was developed in the 1950s and first used as an antidandruff agent in 1961 even though the reason for dandruff was not exactly known at the time [1, 2]. Some factors that can trigger dandruff are environmental factors, hormonal problems, and imbalance in the microbial biome present in the scalp. The most common factor is imbalance in the microbial biome due to the increase in microorganisms of the *Malassezia* genus whose metabolic cycle involves the degradation of fatty acids, generating irritation and hyperflorative activity of the epidermis [3–6].

The mechanism of action that ZnPT follows as an antifungal agent varies and will depend on the microorganism being studied [7, 8]. ZnPT depolarizes the microorganism membrane, preventing the transport of nutrients and energy production [9, 10], and can also increase the amount of copper present in the cell, diminishing the functions of iron-sulfur proteins [11, 12]. In addition to antifungal properties to combat dandruff, ZnPT acts at the cellular level by being cytostatic, regulating the uncontrolled division of scalp cells, and is antiseborrheic due to the sulfur groups in the molecule [13, 14].

Dandruff is a common problem that affects more than 50% of the world population; thus, the number of products to combat dandruff such as shampoos, conditioners, and creams has increased [15]. The Food and Drug Administration (FDA) has registered ZnPT as an active ingredient for over-the-counter (OTC) drugs. Products with ZnPT are divided into two categories: rinse products, where the allowed content is among of 0.3–2.5%, and nonrinse products, where the allowed content is among of 0.1–0.25% [16, 17]. Therefore, ZnPT is a safe active ingredient with no adverse effects reported in humans, except when used for prolonged periods of time where it can cause sensitivity [18–20].

Many analytical techniques exist to determine ZnPT in cosmetic and pharmaceutical products such as atomic absorption spectroscopy, high-performance liquid chromatography (HPLC), and UV/Vis spectrophotometry. However, these analytical techniques have some drawbacks regarding costs, an elaborate sample preparation, and long analysis times due to the complexity of cosmetic products [21–23].

In this study, two methodologies using titrations (complexometric and potentiometric) were validated where the analysis times are much shorter and do not require sample preparation. Since these titrations are faster and cheaper compared to others, they become practical alternatives for routine analyses of high number of samples. Validation was performed following the United States Pharmacopeia (USP) and International Conference on Harmonisation (ICH) guidelines, evaluating the selectivity, linearity, accuracy, precision, and robustness of the methods mentioned above in shampoo samples [24–26].

## 2. Materials and Methods

### 2.1. Reagents and Samples

Secondary standard of zinc pyrithione (Sigma Aldrich), fuming hydrochloric acid (Merck, 37%), ethylenediaminetetraacetic acid (EDTA) solution (Merck, 0.01 M), eriochrome black *T* indicator (Merck), ammonia solution (Merck, 25%), hydrogen peroxide solution (Merck, 30%), potassium chloride (Merck, >99.9%), ammonium chloride (Merck, >99.8%), and Iodine solution (Merck, 0.05 M). The selected samples were three shampoo formulas (S1, S2, and S3) that have zinc pyrithione as a component. Additionally, a placebo of the shampoos analysed was used.

### 2.2. Method

ZnPT determination in shampoo was performed using complexometric and potentiometric titrations. For the complexometric titration, 6.0 g of the sample were weighed and diluted in water (50 mL). Then, 2.5 mL of hydrochloric acid were added while heating and gently stirring for ten min. 0.5 mL of hydrogen peroxide was added, and the mixture was cooled simultaneously. pH was adjusted with an ammonia solution and 2.5 mL of buffer solution (pH = 10). Finally, the sample was titrated with EDTA (0.01 M) using eriochrome black *T* as an indicator. The equivalence point occurred when the color of the solution changes from violet to blue.

For the potentiometric titration, 6.3 g of the sample was weighed and transferred to the cell of the automatic titrator (Mettler Toledo, T70) along with 50 mL of water and 10 mL of fuming hydrochloric acid. The resulting sample was titrated with an iodine solution (0.05 M) where the final point of the titration was indicated by a platinum electrode (Mettler Toledo, DMI 140-SC). Constant stirring was ensured during the titration.

### 2.3. Method Validation

#### 2.3.1. Selectivity

In order to establish method selectivity, the responses obtained for blank solvents, placebo, zinc pyrithione standard solution, and placebo enriched with zinc pyrithione were compared. Moreover, the sample was subjected to various stress conditions such as photolysis (3 h, UV exposure), thermolysis (3 h, heating), and the action of oxidizing agents (1.0 mL, hydrogen peroxide).

#### 2.3.2. Linearity

Linearity of the system and method was evaluated at five concentration levels from 0.2 to 1.4 w/w%. These solutions were prepared percentage by weight in a beaker, mechanically shaken, and sonicated for ten min. System linearity was determined using water as the solvent for the preparation of standard solutions, and method linearity was evaluated using an additive placebo. The criteria evaluated for linearity were the intersection, the slope, and the determination coefficient.

#### 2.3.3. Detection and Quantification Limit

LOD and LOQ values were determined using the linearity curve with the equations LOD = 3.3 *σ*/*S* and LOQ = 10 *σ*/*S,* where *σ* is the standard deviation of y-intercepts of regression lines and *S* is the slope of the line [27]. In addition, the accuracy of the LOQ was obtained.

#### 2.3.4. Precision

Precision was determined through various parameters such as method and system repeatability, intermediate precision, and reproducibility. The system repeatability was evaluated with three ZnPT standard solutions of different concentrations (0.2%, 0.8%, and 1.4%), and method repeatability was evaluated with three shampoo samples. Intermediate precision was evaluated with the results of two analysts in two different days and reproducibility with the results in two laboratories. Results were estimated by calculating the relative standard deviation (RSD%) for each parameter.

#### 2.3.5. Accuracy

Accuracy was determined with three standard solutions of different concentrations, 0.2, 0.8, and 1.4 w/w %, of ZnPT, performing six trials per solution. Accuracy was reported as percent recovery and RSD% which were calculated globally for all concentration levels.

#### 2.3.6. Robustness

Method robustness was evaluated with the experimental design of Youden–Steiner for five variables. Variables used for the complexometric titration were pH, sample weight, heating time, stirring time, and sample resting time. Variables used for the potentiometric titration were HCl volume, sample resting time, titrant addition rate, speed, and stirring time. Eight tests were performed in duplicate changing the conditions mentioned above.

## 3. Results and Discussion

### 3.1. Selectivity

**Figure 1:**
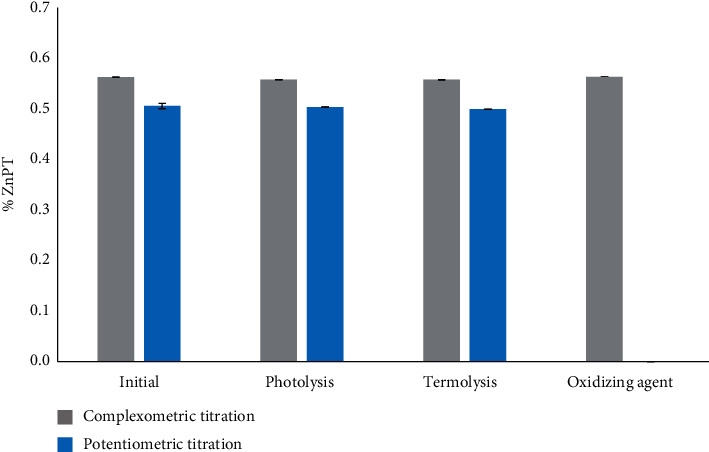
Selectivity of the method under stress conditions. The error bars represent the standard deviation of the replicates made for each working condition (*n* = 3).

The response obtained for solvent blank and placebo was the same, as well as for critical sample and enriched placebo, confirming that the components of the matrix did not interfere in the analysis. The shampoo sample under photolytic and thermolytic conditions did not produce reactions that could interfere with the ZnPT measurement. However, under oxidative conditions, the analyte was not quantifiable for the potentiometric titration, as shown in Figure 1.

### 3.2. Linearity

Values of slope, intercept, and determination coefficient for the studied methods were calculated using the least squares method, and they are shown in Table 1 altogether with t values. Analysis of variance results (ANOVA) confirmed proportionality between the concentration and volume variables, since the F-calculate value is greater than F established in tables. In addition, Student's *t*-test results confirmed a significant linear correlation given that t-calculate is greater than *t* established in the table, which can be verified with the determination coefficient (>0.99).

### 3.3. Sensitivity

LOD and LOQ values were calculated for the system and method of both titrations, obtaining data shown in Table 1, where values were lower for the potentiometric titration. LOQ accuracy was determined as percentage of recovery, generating values among 98% and 101% for the potentiometric and complexometric titrations, these values comply with the limits established for volumetric methods (95–105%) [28]. Additionally, Student's *t*-test results confirmed that no significant difference was found between the recovery percentage values obtained and the theoretical 100%; therefore, the LOQ was accurate in all cases.

### 3.4. Precision

Precision is generally expressed as the RSD% of a series of measurements and expresses conformity or nonconformity regarding the proximity between multiple measurements under specific conditions [27]. RSD% values for repeatability, reproducibility, and intermediate precision were less than 1.0% for the potentiometric and complexometric titration, as shown in Table 2. The values of RSD% were below the limit stipulated according to the Association of Official Analytical Chemists (<5%) [28]; therefore, no significant difference was observed in results of multiple measurements, indicating that both methods are precise and reproducible.

### 3.5. Accuracy

The average recovery of the analytical methodologies was within the limits stipulated for volumetric methods (95–105%) for the active ingredient, as shown in Table 2. The *t* calculated (0.44, complexometric titration and 0.14, potentiometric titration) for the methodologies when compared to the *t* in the table confirmed no statistically significant difference with the value of the average recovery obtained and 100% theoretical; thus, the methodologies were accurate.

### 3.6. Robustness

To determine the robustness of the method, the response obtained by slightly changing the experimental conditions of pH, temperature, stirring time, standing time, and sample weight was evaluated. Effect of these variations was calculated for the two methodologies using the Youden–Steiner formulas and compared with the critical values of 0.0022 and 0.0038 for the potentiometric and complexometric titration [29, 30], as shown in Table 3. The critical value was calculated using equation S ^*∗*^ √2, where *S* is the standard deviation found in the repeatability of the methods. For the potentiometric titration, all values were less than the critical value; therefore, changes in conditions do not significantly affect the method response. For the complexometric titration, the change in the temperature and resting time conditions of the sample differed from the expected responses, suggesting that this methodology was less robust than the potentiometric titration.

## 4. Conclusions

Two simple and practical methods for the quantitative determination of ZnPT in cosmetic products were validated according to USP and ICH guidelines. Validated methods are specific, precise, exact, and linear in the range of concentrations studied. The sensitivity of both methods is adequate, allowing to confidently evaluate the ZnPT content in shampoo samples according to the concentrations established by the FDA. Furthermore, these two methodologies do not require complex sample preparation which makes them suitable techniques for the quality assurance of any shampoo to guarantee the consumer's well being.

## Figures and Tables

**Table 1:** Linearity and sensitivity of the methods studied.

Statistical parameters	Method		System	
	Potentiometric titration	Complexometric titration	Potentiometric titration	Complexometric titration
Regression equation	*y* = 2.5202*x* + 0.0004	*y* = 18.899*x* − 0.0011	*y* = 2.5192*x* − 0.0003	*y* = 18.898*x* − 0.0035
Determination coefficient	1	1	1	1
Standard error of the slope	0.0011	0.0049	0.0002	0.0032
Standard intercept error	0.001	0.0047	0.0002	0.0029
*t*-calculate	2349.9	3877.9	11330	5780.4
Sensitivity (LOD)^*∗*^	0.0013	0.0159	0.0004	0.0159
Sensitivity (LOQ) ^*∗*^	0.0038	0.0534	0.0014	0.0534

^*∗*^% ZnPT.

**Table 2:** Precision and accuracy (*n* = 6) of the method for potentiometric and complexometric titration.

Parameter		Complexometric titration	Potentiometric titration
Intermediate precision RSD%		0.84	0.40
Reproducibility RSD%		0.75	0.31
	0.20%	0.02	0.25
System repeatability RSD%	0.80%	0.03	0.18
	1.40%	0.15	0.10
Method repeatability RSD%	S1	0.47	0.30
	S2	0.70	0.23
	S3	0.69	0.36
Accuracy % recovery		99.89	99.96
Accuracy RSD %		1.06	0.98

**Table 3:** Robustness of the method for potentiometric and complexometric titration.

	Potentiometric titration	Complexometric titration
Critical value	0.0022	0.0038
HCl volume	0.0006	NR^*∗*^
Heating temperature	NR^*∗*^	0.0081
pH	NR^*∗*^	0.0027
Rest time	0.0016	0.0126
Sample weight	NR	0.0223
Starring time	0.0017	0.0018
Starring velocity	0.0014	NR^*∗*^
Titrant addition rate	0.0017	NR^*∗*^

^*∗*^NR: no response.

## Data Availability

The data used to support the findings of this study are available from the corresponding author upon request.
